# Appendicular mass mimicking as suprapubic mass: a case report

**DOI:** 10.4076/1757-1626-2-7815

**Published:** 2009-08-05

**Authors:** Junaid Rafi, Sameer Umranikar

**Affiliations:** Department of Obstetrics and Gynaecology, Basingstoke & North Hampshire NHS TrustAldermaston Road, Basingstoke, RG24 9NAUK

## Abstract

**Introduction:**

We are writing about an unusual case of appendicular mass presenting as painful suprapubic mass in a female patient admitted to gynaecology ward. There has been no recent discussion in the gynaecologic literature of appendicular mass in a young woman presenting as gynaecologic case like this one.

**Case presentation:**

A 39-year-old lady was admitted to gynaecological ward with the complaint of painful suprapubic mass with no bowel symptoms. Subsequent investigations raised the suspicion of tubo-ovarian abscess. The laparotomy revealed burst appendicular mass with involvement of ovaries and part of inflamed bowels. Hence right salpingo-oophorectomy, appendicectomy and right hemicolectomy were performed. The patient made un-remarkable recovery.

**Conclusion:**

Our case presentation highlights the fact that pelvic mass presentation can be misleading, not always of gynaecologic origin therefore clinicians should think broadly as multidisciplinary input may be inevitable.

## Case presentation

A 39-year-old Caucasian (British) non-pregnant lady (para 3 with caesarean section; in a stable relationship) was admitted to gynaecology ward with complain of pelvic pain and temperature of 37.3°C. She was discharged from emergency department three days ago on antibiotics for urinary tract infection. There was no history of vomiting, diarrhoea or constipation. Also there was no past history of pelvic inflammatory disease. The laboratory findings were CRP 443 mg/L; Hb 111 g/L; WBCs 11.9 × 10^9^ /L with 83% neutrophilia and Platelets were 386 × 10^9^ /L. Urine dip stick revealed no infection. The abdominal examination revealed a fixed firm immobile suprapubic mass with mild tenderness but no signs of peritonism. The uterus was bulky, mobile and adnexal mass noted on bimanual examination. Her cervical smears were up to date and there was no family history of ovarian, cervical or endometrial carcinoma. Her CA-125 was also within normal limits.

**Figure 1.**
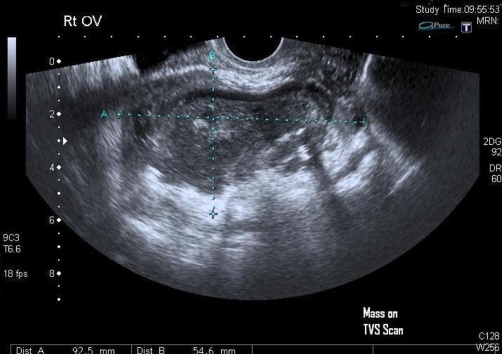
Mass seen on transvaginal scan.

**Figure 2.**
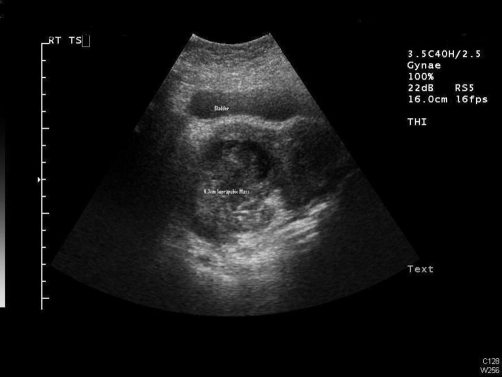
Bladder and Mass can be seen on abdominal scan.

The ultrasound transvaginal (Figure 1) and ultrasound abdomino-pelvis (Figure 2) revealed right adnexal lesion measuring 9.3 × 8.3 × 6.6 cm with mixed echogenicity with small fluid areas as well as some focal echogenic areas consistent with some air or gas. There was some peripheral vascularity but no central vascularity on dopplers. No free fluid in pouch of Douglas noted and no abnormality seen in right iliac fossa. The appearances were most likely to represent a right tubo-ovarian abscess. The radiologist recommended no further investigations. Therefore an exploratory Laparotomy was performed and it revealed burst appendix, faecolith matter; tubes and ovary buried in inflamed mass on right side. Approximately 200 ml pus was drained from the area between posterior of uterine fundus and inflamed bowels. The gynaecology team performed right salpingo-oophorectomy while appendicectomy with right hemicolectomy was performed by surgeons. The patient made un-remarkable recovery.

## Discussion

There has been no recent discussion in the gynaecologic literature of appendicular mass in a young woman presenting as gynaecologic disease like this case. Because of the proximity of the right ovary to the appendix, it is possible for appendicitis to perforate into the ovary, producing a tubo-ovarian abscess indistinguishable from that due to other more common causes.

Jinxing Yu et al [1] in a very comprehensive pictorial (Helical CT scan) essay described the un common mimics of appendicitis which can present as gynaecologic disease are ovarian vein thrombosis, ovarian dermoid, necrotic uterine leiomyoma, ovarian torsion, endometriosis and ruptured ectopic pregnancy. The gastrointestinal processes such as diverticulitis and appendiceal abscess can present as pelvic mass but presentation as suprapubic mass is quite unusual and not reported in literature before. This rare presentation of appendicular mass alerts gynaecologists as well as surgeons, highlighting the needs of lateral thinking and multidisciplinary input.

## Conclusion

In a sexually active female, the presentation of pelvic pain is commonly dealt as a gynaecologic symptom as all the emphasis is ruling out common causes like ovarian accidents and pelvic inflammatory disease. Appendicitis though important differential diagnosis is often missed in reproductive age group females. In this case the pelvic pain turned out to be a misleading symptom with relevance to gynaecologic disease instead was a more acute abdominal presentation requiring urgent intervention. It was an unusual presentation for appendicular mass, highlighting the importance for gynaecologists for lateral thinking when seeing pelvic mass patients especially in younger females with low risk of malignancy. In this unusually presenting patient there was higher risk missed diagnosis and of complications as typical signs of acute appendicitis, tenderness at Mac Burney’s point and peritonism were absent. Delayed diagnosis led to eventual loss of ovary and tube, and this can have detrimental effect on woman’s life both physically and psychologically especially if she has not completed her family. Consequently on reflection our case presentation highlights the fact that pelvic mass presentation can be misleading, not always of gynaecologic origin therefore clinicians should think broadly as multidisciplinary input may be inevitable.

## Abbreviations

CA-125: Cancer antigen 125
CRP: C reactive protein
Hb: Haemoglobin
WBC: white cell count
